# Paternal UPD (15) With Disease-Causing Mutation and Small Supernumerary Ring Chromosome 15: A Case Report

**DOI:** 10.1155/crig/4973753

**Published:** 2025-07-25

**Authors:** David Lee Curtis, Nasim Bekheirnia, Lorraine Potocki, Ludmila Matyakhina, Mir Reza Bekheirnia

**Affiliations:** ^1^Baylor College of Medicine, Houston, Texas, USA; ^2^Texas Children's Hospital, Houston, Texas, USA; ^3^Department of Pediatrics, Division of Pediatric Nephrology, Baylor College of Medicine, Houston, Texas, USA; ^4^Department of Molecular and Human Genetics, Baylor College of Medicine, Houston, Texas, USA; ^5^GeneDx, Gaithersburg, Maryland, USA

**Keywords:** Angelman syndrome, Bartter syndrome, mosaicism, ring chromosome 15, uniparental disomy, UPD

## Abstract

Uniparental disomy (UPD) constitutes an unconventional mode of inheritance that disrupts the typical biparental genetic contribution and may result in phenotypic abnormalities. This report centers on a patient diagnosed with Bartter syndrome Type 1, attributed to a homozygous pathogenic variant in *SLC12A1* unmasked by mosaic paternal UPD of chromosome 15. We hypothesize that this pattern (or constellation) emerged from a trisomy rescue event, resulting in two distinct cell lines. Concurrently, the unmasking of a pathogenic paternal *SLC12A1* variant by trisomy rescue resulted in the manifestation of Bartter syndrome Type 1. The maternally derived ring chromosome 15 and its impact on nondisjunction and UPD elucidate a unique etiology of Bartter syndrome. Furthermore, the presence of a pathogenic paternal *SLC12A1* variant underscores the pivotal role of trisomic rescue and paternal UPD in unveiling a recessive variant.

## 1. Introduction

Uniparental disomy (UPD) represents an uncommon genetic phenomenon where both chromosomes of a given pair are inherited from one parent, leading to the absence of the other parent's contribution for that pair. This atypical mode of inheritance can give rise to phenotypic abnormalities due to differences in gene expression based on parental origin [1, 2]. Additionally, the presence of uniparental isodisomy can result in two copies of a recessive allele from a single parent, which may lead to the manifestation of recessive disorders that would otherwise remain unexpressed [3].

UPD often arises from errors in chromosomal separation during meiosis of parental germline cells, a process known as nondisjunction. These events can generate abnormal gametes containing either two copies of a chromosome (disomic) or no copies of that chromosome (nullisomic), deviating from the normal haploid state of one copy per chromosome. Fertilization with one of these nondisjunction products results in either a monosomic or trisomic zygote. A subsequent event, involving loss of a chromosome in the trisomy case or duplication of a chromosome in the monosomy case, can occur, leading to the restoration of a karyotypically normal status termed UPD [4].

Various postfertilization mechanisms, including mitotic recombination resulting in partial isodisomy and postfertilization nondisjunction events necessitating an additional rescue event, can also lead to UPD, depending on the timing of the nondisjunction event [3, 5]. As most nondisjunction events occur within maternal meiosis I, the majority of nullisomic and disomic gametes tend to be derived from maternal oocytes [6]. Consequently, UPD derived from a trisomic rescue will typically be maternally heterodisomic, while UPD from monosomic rescue will be paternally isodisomic [7].

The phenotypic impact of UPD varies among different chromosomes. While most genes do not exhibit noticeable phenotypic effects, a select few are subject to parent-specific imprinting, leading to clinically recognizable consequences. Specifically, maternally derived genes on chromosomes 7, 14, 15, and 20, as well as paternally derived genes on chromosomes 6, 11, 14, 15, and 20, have been definitively shown to manifest phenotypic effects due to uniparental inheritance of imprinted regions [4, 5, 8].

Supernumerary chromosomes, including ring chromosomes, have been shown to be associated with chromosomal instability rather than nondisjunction [9]. Ring chromosomes, which are mitotically unstable altered chromosome products, are generally believed to result from the de novo breakage of both chromosomal end-segments followed by subsequent fusion to form a ring. They are heterogeneous in terms of size, chromosomal origin, association with deletions, and, due to their instability and frequent occurrences of loss, fragmentation, or translocations, they display highly variable phenotypes [10].

The chromosome 15 region q11–q13 is imprinted and contains genes expressed only from the paternally or maternally inherited chromosome [11]. Deletions in this region on the maternal chromosome cause Angelman syndrome (AS) (OMIM #105830), while on the paternal chromosome they cause Prader-Willi syndrome (PWS) (OMIM #176270) [12, 13]. These conditions can also result from UPD of chromosome 15. Duplication of the Prader-Willi/Angelman syndrome critical region (PWACR) can also lead to an abnormal phenotype (OMIM #608636), particularly when inherited maternally [14, 15]. These extra copies can manifest as interstitial duplications/triplications or as (r(15)).

Bartter syndrome (BS), first identified in 1962 by Bartter et al., comprises a group of autosomal recessive disorders that impact salt reabsorption. It is characterized by hypokalemic metabolic alkalosis, significant salt wasting, low or normal blood pressure, hypercalciuria, and secondary hyperaldosteronism [16, 17]. Type I BS (OMIM #601678) is attributed to a defect in the *SLC12A1* gene located on 15q21.1, which consists of 26 exons and encodes the sodium/potassium/chloride transporter (*NKCC2*) [18, 19].

## 2. Materials and Methods

### 2.1. Patient Accrual

Patient was enrolled in an institutional review board-approved research study. The procedures followed were in accordance with the ethical standards of the institution's committee on human research and were in keeping with international standards.

### 2.2. Chromosomal Microarray Analysis and Exome Sequencing (ES)

Chromosomal microarray analysis was performed on a clinical basis through GeneDx using a 2–5 mL blood sample in EDTA. ES was performed through AiLife Diagnostics using exome capture and Next-Generation Sequencing (NGS). The sample was collected through saliva.

### 2.3. Case Report

The proband is a 35-year-old female who presented with polycystic ovary, short stature, mild intellectual disability, ring chromosome 15, and a previous diagnosis of diabetes insipidus. She was conceived naturally, and mother's pregnancy was complicated by polyhydramnios which was treated with amniocentesis and Indomethacin [20, 21]. At that time, chromosome analysis of the amniotic fluid was performed. The proband was delivered via spontaneous vaginal delivery (SVD) at 35 weeks, weighing 5 pounds. Following birth, she spent 8 days in the neonatal intensive care unit (NICU) before being discharged home with her parents.

At 35 weeks of gestation, a chromosome study of amniotic fluid revealed the presence of a marker chromosome which, through repeat chromosome analysis at age 12, was determined to be a ring chromosome 15. High-resolution Chromosomal analyses of both parents were normal. According to the mother's report, at 8 months of age, the patient weighed only 10 pounds, indicating difficulties with weight gain. Additionally, she demonstrated short stature, being just below the fifth percentile for her age, yet with a normal growth velocity. Subsequently, the patient underwent evaluation by a gastroenterologist, who identified high sodium and low potassium levels along with low urine gravity. Based on these findings, the patient had received a clinical diagnosis of diabetes insipidus at that time.

In addition to these findings, the proband also exhibited premature adrenarche of unknown etiology, experiencing the development of pubic and axillary hair at age 7, which did not progress further. Her motor development was normal, but she had speech delay, necessitating therapy from ages 3 to 5. IQ testing conducted at age 7 revealed an IQ range between 80 and 90. The patient has no craniofacial dysmorphology and no cardiac abnormalities.

## 3. Results

Clinical genetic testing was performed using trio ES and comprehensive CMA with both oligonucleotide probes and SNPs. The trio ES results revealed a homozygous likely pathogenic variant based on ACMG/AMP guidelines in the paternally inherited *SLC12A1* gene (the mother tested negative for this variant) confirming a diagnosis of BS Type 1. This was a change in the previous suspicion of diabetes insipidus. The CMA result was also positive, indicating a mosaic copy number gain involving cytogenetic bands 15q11.2 to 15q13.3 in approximately 70% of the patient's cells. Furthermore, the CMA result indicated that the proband carries two homologous copies of chromosome 15, which share identical sequences spanning from cytogenetic band 15q11.2 to 15q26.3, encompassing the entire length of the chromosome's long arm. Methylation-specific MLPA testing confirmed an abnormal imprinting pattern consistent with the predominant contribution of paternal chromosome 15. Fluorescence in situ hybridization (FISH), using whole chromosome painting specific for chromosome 15 showed the ring chromosome to be of chromosome 15 origin.

The proband presented here has a de novo, mosaic ring-chromosome 15, present in approximately 70% of cells, along with a molecular diagnosis of BS Type 1, likely due to a homozygous variant in the *SLC12A1* gene. Chromosome microarray analysis shows that the ring includes the PWACR. The remaining 30% of the patient's cells contain pure paternal UPD, likely arising from a monosomy rescue event.

Trio ES testing revealed the presence of a homozygous variant, c.1560+1G > A in the *SLC12A1* gene. This variant was not found in the mother but was identified as heterozygous in the father. According to the ACMG/AMP guidelines, this variant is interpreted as a likely pathogenic variant of the *SLC12A1* gene, confirming the clinical diagnosis of BS Type 1. The variant has a reported frequency of 2.4e05 in the gnomAD database.

The CMA result, which was confirmed using methylation-specific MLPA (MS-MLPA), showed the presence of a 10.68 Mb copy number gain extending from cytogenetic band 15q11.2 to 15q13.3. Additionally, an abnormal methylation pattern was observed in 15q11.2. This copy number gain is present in approximately 70% of cells and is consistent with the previously identified ring chromosome 15. Cytogenetic analysis revealed a female karyotype with a small supernumerary marker chromosome identified as a ring chromosome derived from chromosome 15, present in mosaic form: 47, XX, +r(15) (p13q13.3). The duplicated interval includes the critical region for Prader-Willi and ASs as well as the recurrent 15q13.3 microdeletion/microduplication region, extending from BP1-BP5.

Furthermore, a region of homozygosity was identified, extending from cytogenetic band 15q11.2 to 15q26.3, which encompasses the entire long arm of the chromosome. MS-MLPA testing confirmed an abnormal paternal imprinting pattern, indicating a predominant contribution of the paternal chromosome 15. The microarray and MLPA data together suggested the presence of two cell lines: one predominant line (70% of cells) with two copies of the paternal chromosome 15 and a gain of proximal 15q due to the presence of a (r(15)) (p13q13.3), and a second line (30% of cells) with only two copies of the paternal chromosome 15, consistent with paternal UPD15; again, supporting the ES data. Considering the MS-MLPA data and prior karyotype results, these array data also support the presence of a maternally derived mosaic chromosome.

**Figure 1:**
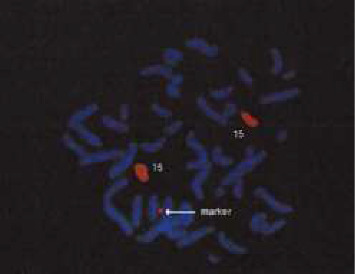
Image depicting the FISH analysis of the proband using painting probe for chromosome 15.

Lastly, FISH had also been conducted and confirmed the ring chromosome to be of chromosome 15 origin. A painting probe specific to other chromosomes did not hybridize to the marker (Figure 1).

## 4. Discussion

In this case report, we provide a thorough analysis of a patient exhibiting BS Type 1, polycystic ovary, short stature, and mild intellectual disability. Our primary focus is on understanding the intricate interplay among a maternally derived ring chromosome 15, paternal UPD of chromosome 15, and a homozygous *SLC12A1* variant inherited on the paternal chromosome 15. Our discussion aims to elucidate the potential relationships between these genetic findings and their individual and collective contributions to the phenotype of the proband.

We hypothesize that the ring chromosome likely originated within the maternal gamete before fertilization. Upon fertilization of the oocyte containing the ring by a monosomic sperm, a monosomic rescue duplication of the paternal chromosome 15 was likely necessary for the survival of the conceptus. Subsequent cellular replications likely led to mitotic instability induced by the presence of the ring, culminating in a nondisjunction event. This event resulted in the loss of the ring and the emergence of two distinct cell lines: a predominant cell line containing two paternal chromosome 15s with the maternal ring (15) and a minor cell line containing only the two paternal chromosome 15s without the ring (15).

Furthermore, the presence of a recessive, disease-causing variant of the *SLC12A1* gene on the paternal chromosome 15 likely contributed to the development of BS in the proband. The process of monosomy rescue apparently played a critical role in unmasking the homozygous state of this variant, thereby leading to the observed phenotype of BS Type 1.

Regarding the patient's phenotype, the UPD (30%) apparently does not exhibit an AS causing methylation defect due to the contribution from the maternal structural abnormality present in the predominant cell line (70%). Given the strong predominance of this chromosome 15 duplication cell line, we anticipate that its corresponding phenotype would exert a greater influence on the proband's phenotype compared to the UPD cell line with AS methylation pattern (30%).

The presence of moderate intellectual disability, speech delay without speech absence, and the relative scarcity of other clinical features of AS, the phenotype of this patient appears to align more closely with the chromosome 15 duplication phenotype rather than AS. However, in mosaic cases, phenotype manifestation may be influenced by tissue distribution.

In conclusion, our findings highlight the intricate genetic mechanisms involved in the proband's condition, including the co-occurrence of BS, ring (15) and paternal UPD (15). The maternal origin of the ring chromosome 15 and its subsequent impact on nondisjunction and UPD provide insights into a unique etiology of BS in this individual. Additionally, the presence of a pathogenic paternal *SLC12A1* variant underscores the role of monosomy rescue and paternal UPD in unmasking recessive variants.

To date, at least 15 other cases of UPD (15) with pathogenic mutations due to isodisomy have been reported, involving both maternally and paternally derived chromosomes (https://cs-tl.de/DB/CA/UPD/15-UPDm.html; https://cs-tl.de/DB/CA/UPD/15-UPDp.html). In each of these cases, homozygosity of an autosomal recessive pathogenic variant was facilitated by segmental or full uniparental isodisomy. Our patient represents a novel addition to this cohort, with a homozygous *SLC12A1* variant arising from paternal uniparental isodisomy of chromosome 15. Importantly, our case also includes a mosaic small supernumerary marker chromosome, characterized as a ring [r(15)], which has not been previously described in this context. This combination of UPD and a structural chromosomal anomaly illustrates how chromosomal rescue mechanisms can unmask recessive disease in atypical presentations.

## Data Availability

Data sharing is not applicable to this article as no datasets were generated or analysed during the current study.
